# Positional specialization of LIR motifs in RavZ and the autophagy-related protein ATG4B

**DOI:** 10.1080/27694127.2024.2438563

**Published:** 2024-12-19

**Authors:** Sang-Won Park, Jin-A Lee, Deok-Jin Jang

**Affiliations:** a Department of Vector Entomology, College of Ecology and Environment, Kyungpook National University, Sangju, Korea; b Department of Biological Sciences and Biotechnology, College of Life Sciences and Nanotechnology, Hannam University, Daejeon, Korea; c Research Institute of Invertebrate Vector, Kyungpook National University, Sangju, Korea

**Keywords:** Autophagy, LC3/GABARAP, ATG4B, RavZ, delipidation, LIR

## Abstract

LC3-interacting region (LIR) motifs are essential for recruiting proteins onto autophagosomes, the hallmark of autophagy. We recently explored the relevance of the specific position of the LIRs in RavZ and ATG4B (autophagy-related 4B). RavZ’s N-terminal LIRs drive substrate recognition and enzymatic activity, while its C-terminal LIR aids membrane localization. In contrast, ATG4B’s C-terminal LIR is indispensable for LC3B (microtubule-associated protein 1 light chain 3B)-phosphatidylethanolamine (PE) delipidation on autophagosomes but not required for cytosolic LC3B priming, which is mediated solely by its catalytic domain (CAD). These findings underscore the structural adaptation of LIRs for context-specific functions. This novel nuanced understanding provides a framework for developing therapeutic tools to modulate autophagy by precisely targeting LIRs or their associated processes, offering potential treatment for diseases like neurodegenerative disorders and infections characterized by autophagy dysregulation.

## Text

Autophagy, an intracellular degradative process, relies on precise recruitment of proteins to autophagosomes via interactions with members of the LC3/GABARAP (gamma-aminobutyric acid receptor-associated protein). This recruitment is mediated by LIRs, short amino acid sequences that bind to the hydrophobic pocket at the N-terminus of LC3/GABARAPs. While the functionality of LIRs has been extensively studied, the relevance of their position within proteins remains poorly understood. Recently, we investigated the roles of N- and C-terminal LIRs in RavZ, a *Legionella pneumophila* effector, and ATG4B, a mammalian cysteine protease essential for autophagy [1].

**Figure 1.**
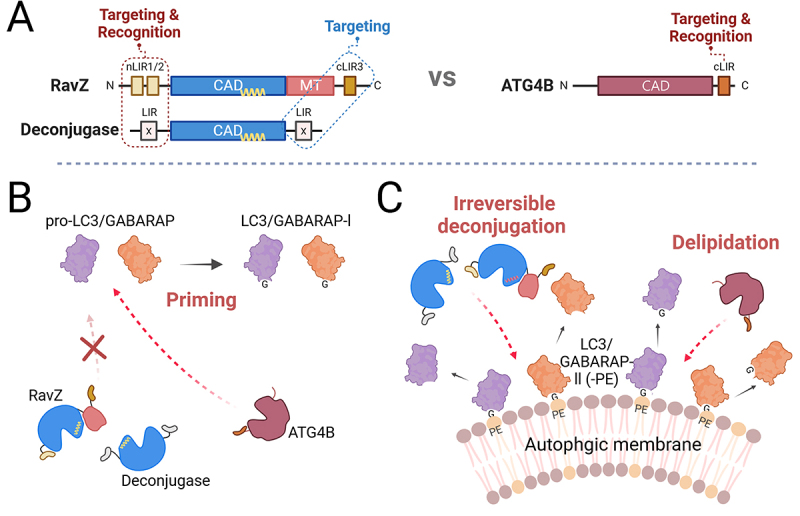
Role of RavZ and ATG4B LIRs in Autophagy Pathway (A) The N-terminal LIR of RavZ and the C-terminal LIR of ATG4B are critical for targeting LC3/GABARAP and enabling the catalytic activity of their respective CADs. Additionally, RavZ’s C-terminal LIR aids in binding LC3/GABARAP-PE on autophagosomes. (B) RavZ cannot cleave LC3/GABARAP in the cytosol; at autophagosomes, its N-terminal LIR recognizes LC3/GABARAP for irreversible cleavage. (C) While ATG4B’s C-terminal LIR is unnecessary for cytosolic LC3/GABARAP priming, it is essential for LC3/GABARAP delipidation at autophagosomes.

RavZ has an MT (membrane targeting) domain and three LIRs, two N-terminal and one C-terminal. Although the MT domain of RavZ plays a significant role in membrane localization, experiments with MT-deficient RavZ demonstrated that LIRs are indispensable for guiding the protein to autophagosomes and facilitating the delipidation of LC3B-PE. RavZ N-terminal LIRs primarily drive substrate recognition and catalytic efficiency (Figure 1). Mutational analysis revealed that RavZ constructs lacking these motifs exhibited significantly reduced ability to delipidate LC3B-PE from autophagosomes. This highlights their indispensable role in orienting the CAD for effective substrate processing. The C-terminal LIR, in contrast, facilitates membrane localization by tethering RavZ to autophagosomes. Although both N- and C-terminal motifs contribute to membrane targeting, the C-terminal LIR plays a supplementary role, ensuring proper spatial positioning of RavZ on autophagosomes (Figure 1). Unlike the N-terminal motifs, its impact on catalytic activity is minimal.

These findings underscored the positional s..pecialization of LIRs in RavZ. While the N-terminal motifs are pivotal for substrate interaction and enzymatic function, the C-terminal motif optimizes membrane association. Together, these motifs enable RavZ to effectively disrupt host autophagy by selectively recognizing and irreversibly cleaving LC3B-PE. In full-length RavZ, its function relies more on the MT domain than the LIRs. The MT domain enables stable membrane association, allowing *Legionella* to target all seven LC3/GABARAPs simultaneously, which likely explains the reduced binding strength of the N-terminal LIRs.

The C-terminal LIR of ATG4B is crucial for delipidating LC3B-PE from autophagosomes. Mutants lacking this motif are completely unable to delipidate LC3B-PE, underscoring its indispensable role in substrate recognition. Without the LIR or artificial insertion of the LIR into N-terminus, ATG4B fails to engage effectively with LC3B-PE on autophagosomes, disrupting the autophagic flux. Interestingly, the CAD of ATG4B alone is sufficient to cleave pro-LC3B into LC3B-I in the cytosol, a process independent from ATG4B LIR. This finding delineates a clear separation of function: while the CAD autonomously primes LC3B, the C-terminal LIR is exclusively required for the delipidation of LC3B-PE (Figure 1). In addition, it does not appear to function when the LIR is located at the N-terminus. Even if a functional LIR site is present at the N-terminus, it seems to have lost its functionality, at least in autophagy, likely because it is no longer essential for its role. It cannot be excluded that LC3s are also present on other membranes, and this LIR may be involved in pathways beyond autophagy. However, the C-terminal LIR plays a critical role in tethering ATG4B to autophagic membranes and enabling interaction with lipidated substrates, a prerequisite for effective LC3/GABARAP recycling. In contrast, the cytosolic priming of pro-LC3B to LC3B-I is entirely reliant on CAD, independent of the LIR. This duality highlights a structural optimization in ATG4B that allows distinct processes — membrane-associated delipidation and cytosolic priming — to occur without interference.

The four members of the mammalian ATG4 protein family, ATG4A to ATG4D, share functional roles in autophagy but differ in LIR positioning and substrate specificity. While ATG4B has a well-characterized C-terminal LIR essential for membrane interactions, the LIRs in ATG4A, ATG4C, and ATG4D are less studied and may vary in function and position. In other organisms, such as yeast and plants, ATG4 proteins either lack a conserved LIR or have it in a different position, reflecting evolutionary adaptation to their specific autophagic substrates and machinery. This divergence likely arises from functional specialization, structural constraints, and organism-specific requirements for substrate targeting or membrane localization.

This study reveals the unique roles of LIRs, challenging their perceived redundancy and offering a foundation for engineering proteins to modulate autophagy. Thus, this study provides a conceptual framework for designing therapies that leverage the positional specialization of LIRs in RavZ and ATG4B. For neurodegenerative diseases such as Alzheimer’s and Parkinson’s, where defective autophagy contributes to the accumulation of damaged proteins and organelles, mimicking ATG4B’s C-terminal LIR could enhance LC3B-PE delipidation. This would improve autophagic flux and promote the clearance of cellular debris. Conversely, in infections caused by pathogens like *Legionella pneumophila*, competitive inhibitors targeting RavZ’s N-terminal LIRs could block its interaction with LC3/GABARAPs, preserving the host’s autophagic defenses. Such approaches offer precise therapeutic strategies to restore or regulate autophagy in disease contexts.

Beyond therapeutic applications, positional insights into LIRs can drive innovation in biotechnology. Synthetic biology could utilize engineered LIRs to direct therapeutic proteins or enzymes to autophagosomes, enhancing their degradation efficiency or specificity. For instance, adding an N-terminal LIR to therapeutic enzymes could improve their autophagic targeting. Additionally, engineered ATG4B variants with modified LIRs could be employed in industrial processes, such as optimizing autophagy in cell lines for protein production or organoid development. Furthermore, these insights could allow high-throughput screening platforms to discover small molecules that modulate autophagy, accelerating drug discovery for autophagy-related diseases. These applications highlight the potential for LIR engineering to bridge gaps between basic research, therapeutic development, and industrial biotechnology.

## Abbreviations

ATG4B: autophagy-related 4B
CAD: catalytic domain
GABARAP: gamma-aminobutyric acid receptor-associated protein
LC3B: microtubule-associated protein 1 light chain 3B
LIR: LC3-interacting region
MT: membrane targeting
PE: phosphatidylethanolamine.
